# Coapting the non-regurgitant A2P2 segment effectively reduced atrial functional mitral regurgitation with eccentric jets

**DOI:** 10.1093/ehjcr/ytae542

**Published:** 2024-09-30

**Authors:** Zhen-Gang Zhao, Yu-Jia Liang, Yuan Feng, Mao Chen

**Affiliations:** Department of Cardiology, West China Hospital, Sichuan University, 37 Guoxue Road, 610041 Chengdu, China; Department of Cardiology, West China Hospital, Sichuan University, 37 Guoxue Road, 610041 Chengdu, China; Department of Cardiology, West China Hospital, Sichuan University, 37 Guoxue Road, 610041 Chengdu, China; Department of Cardiology, West China Hospital, Sichuan University, 37 Guoxue Road, 610041 Chengdu, China; Laboratory of Cardiac Structure and Function, Institute of Cardiovascular Diseases, West China Hospital, Sichuan University, 37 Guoxue Road, 610041 Chengdu, China

**Figure:**
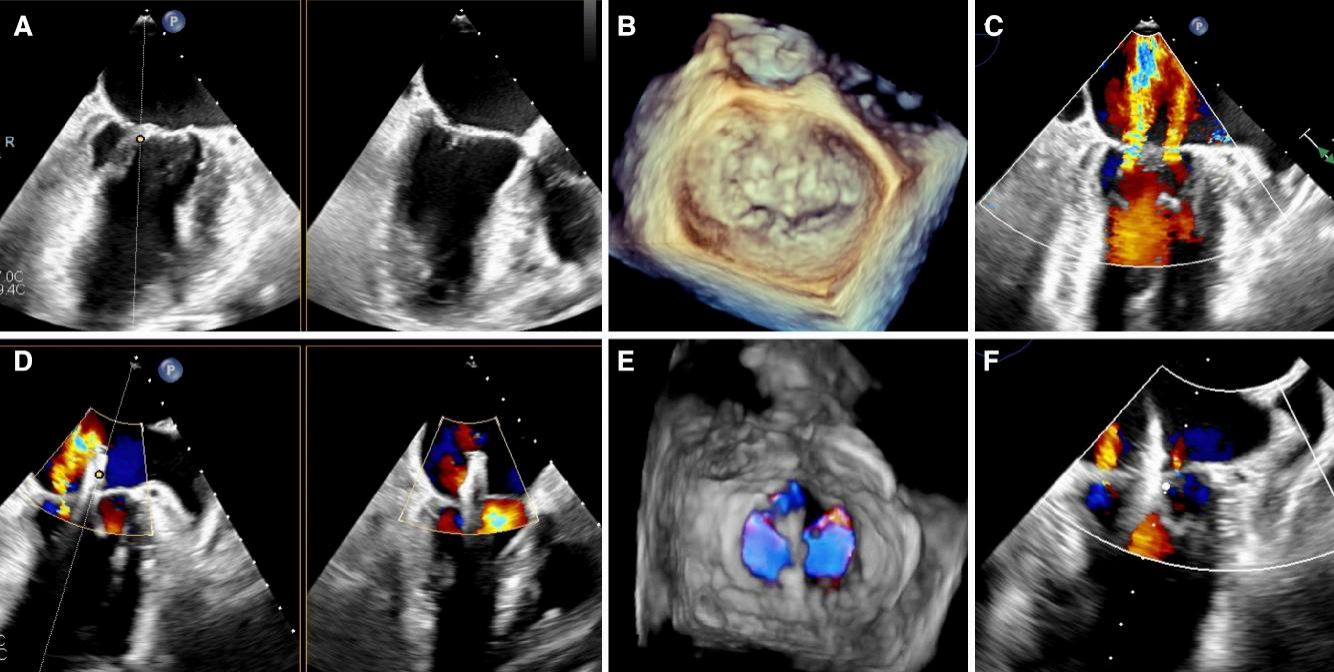


A 77-year-old female with a 14-year history of atrial fibrillation was re-hospitalized for acute decompensated heart failure. Transoesophageal echocardiography (TEE) showed moderate-to-severe (3+) mitral regurgitation (MR) with reduced and flattened leaflet coaptation (*Panels A and B*; Supplementary material online, *Video S1*), suggesting atrial functional MR (AFMR). Mitral regurgitation originated from A1P1 (vena contracta = 4 mm) and A3P3 (vena contracta = 5 mm) segments (effective regurgitant orifice area = 0.38 cm^2^, regurgitant volume = 55 mL), separated by a 6 mm regurgitant-free region at A2P2 (*Panel C*; Supplementary material online, *Video S2*). Posterior leaflet length was 8.5 mm at A2P2 but <7 mm at the regurgitant segments. The mitral valve area was 4.7 cm^2^.

She was considered a reasonable candidate of transcatheter edge-to-edge repair (TEER) despite technical challenges. The routine practice of mitral TEER was to grasp the leaflet segment with maximal regurgitant jet. However, considering the short posterior leaflet outside A2P2, and the emergence of new significant jets when clipping directly at the eccentric regurgitant jets, we decided to intentionally coapt the non-regurgitant A2P2 (CONAP2) segment. Surprisingly, the CONAP2 strategy with a single MitraClip XTR (*Panels D and E*; Supplementary material online, *Video S3*) resulted in significant overall MR reduction to trivial (*Panel F*; Supplementary material online, *Video S4*) with acceptable transmitral gradient (4 mmHg). Mitral regurgitation reduction could be the combined effect of coaptation enhancement and reduced anteroposterior annular diameter (from 39 to 34 mm) and consequent increase in the leaflet-to-annulus index.

Atrial functional MR is a well-characterized subtype of FMR with reportedly satisfactory response to TEER; however, the optimal strategies remain to be defined, particularly in complex cases.^1–3^ Multiple-clip strategy may be effective for AFMR with multiple eccentric jets, but would be difficult in cases with relatively small mitral valve area and short posterior leaflet. We demonstrated that TEER with the CONAP2 strategy regardless of the regurgitant jet location could be effective in MR reduction in the setting of AFMR with multiple eccentric jets. We believe that long-arm clip should be an important element of the CONAP2 strategy for sufficient coaptation enhancement and anteroposterior annular dimension reduction. Nevertheless, future studies are needed to confirm our findings.

## Supplementary Material

ytae542_Supplementary_Data

## Data Availability

The data underlying this article are available in the article and in its online Supplementary material.

## Supplementary material

**Consent:** The authors confirm that written consent for the submission and publication of this case report has been obtained from the patient in line with the COPE guidelines.

**Funding:** The work was supported by Sichuan Science and Technology Program (grant numbers 2022ZDZX0020 and 2022ZDZX0030), Sichuan Provincial Cadre Health Research Program (grant number ZH2024-103), and the 1·3·5 Project for Disciplines of Excellence—Clinical Research Fund, West China Hospital, Sichuan University (grant number 2024HXFH038).
